# Vandenbroucke and Pearce Respond to “Incident and Prevalent Exposures and Causal Inference”

**DOI:** 10.1093/aje/kwv219

**Published:** 2015-10-26

**Authors:** Jan Vandenbroucke, Neil Pearce

We are grateful for the opportunity to reply to the comments by Hernán (1) and Brookhart (2) on our paper about incident and prevalent exposures and causal inference, in which we argued that limiting studies to persons who are followed from first exposure onward may damage epidemiology (3).

In an imaginary world, the ideal study would start from first exposure and follow all study participants until the end of their lives. In the real world, the issue is not *whether* there will be missing data for some time periods. There always will be. Rather, the issue is *which* time periods we should focus on. The question is not whether “left truncation is okay” but whether “left truncation or right censoring is a more serious problem in *this* study of *this* hypothesis.” It is not a matter of idealism versus pragmatism but which type of pragmatism is most appropriate in a given situation. In a study of alcohol consumption and car accidents, missing data on the 24 hours after drinking (i.e., left truncation) would be disastrous. In a study of occupational cancer, the first 5 years after exposure may not be of interest, whereas data for the period of more than 10 years after first exposure are crucial.

Hernán's analogy that we are complacent about seat-belt use because risks are small (1) is misleading. The issue is not about wearing a seat belt but whether “not wearing a seat belt” is the most serious problem. When taking an airplane, would you rather 1) wear a seat belt during takeoff, 2) know that the plane had enough fuel to reach its destination, or 3) know that the landing gear worked? The ideal trip has all 3, and the ideal epidemiologic study has perfect and infinite follow-up. In the real world, however, we need to make choices: We are almost always faced with accepting left truncation, right censoring, or both, and the best decision depends on the hypothesis under study.

Fundamentally, we disagree when Hernán sees the purpose of epidemiologic research as specifically to support practice and limits epidemiology to studies that resemble randomized trials. We conduct epidemiologic studies for many reasons, including to assess causality for factors that will not lead to immediate interventions, that is, to explore etiology. Many studies involve complex exposures (e.g., socioeconomic status, climate change) that are relevant to public health policy but do not correspond to a randomized trial, even in theory. Hernán chooses to define epidemiology in a narrow way because this fits the elegant (and often incredibly useful) randomized trial-based theory that he advocates. This ultimately means restricting epidemiology to studying the short- or medium-term effects of very specific interventions and not studying many important public health problems (has there ever been an incident-exposures study of smoking and lung cancer or of a gene and cancer at middle age?). However much we admire Brookhart's ideas about the treatment decision design (2) and hope that this might be applied in pharmacoepidemiology, it does not solve the problems we raise.

What are the rest of us supposed to do? Should we tell society, “Sorry, we can't give you answers, or even evidence, about major scientific issues because of ‘left truncation’?” Should we throw out all knowledge on the adverse effects of drugs on the basis of periods of use (oral contraceptives, nonsteroidal antiinflammatory drugs), even if it is clear that the adverse effect occurs only during use and is unlikely to be modified by time since first use?

Whether a study is based on incident or prevalent exposures, it is important to document this and to carefully assess the likely biases. What we have is a choice between different types of pragmatism: Which type works best depends on the hypothesis under study and the populations available to study it. What we need is problem-based methods rather than theory that tautologically infers that some study designs are inherently and always better than others.
